# Evolution of self-reactive B cells: lessons from a lupus model

**DOI:** 10.1042/BST20250392

**Published:** 2026-07-31

**Authors:** Kristine Oleinika, Michael C. Carroll

**Affiliations:** Program in Cellular and Molecular Medicine, Boston Children’s Hospital, Harvard Medical School, Boston, MA, U.S.A.

**Keywords:** B cells, epitope spreading, self-reactive, systemic lupus erythematosus

## Abstract

Systemic lupus erythematosus develops when autoreactive B cells escape tolerance and enter differentiation pathways that sustain pathogenic autoantibody responses. A defining feature of lupus is the evolving autoantibody repertoire, in which initially focused autoreactivity broadens over time through recruitment of additional self-reactive B cell clones as well as continued mutation and selection of B cells engaged in the response. Here, we review insights from the 564Igi lupus model, in which a defined autoreactive B cell clone creates an autoimmune environment that permits a previously tolerant polyclonal wild-type (WT) B cell repertoire to enter the response. Studies using mixed bone marrow chimera and adoptive transfer approaches show that WT-derived B cells can be recruited into established autoreactive niches, participate in germinal center and extrafollicular pathways, and contribute to epitope-spread autoantibody responses. We discuss how studies in the present model have reframed epitope spreading as a dynamic process of self-reactive B cell evolution, in which otherwise restrained B cells are incorporated into an ongoing autoimmune response and shaped by the niches that support their activation, differentiation, persistence, and diversification.

## Lupus as a failure of B cell tolerance

Systemic lupus erythematosus (SLE) is a chronic autoimmune disease in which self-reactive B cells become activated and produce autoantibodies that contribute to tissue inflammation and organ damage [1,2]. This represents a breakdown of B cell tolerance—the failure to maintain control over B cells with specificity for self-antigens.

The clinical manifestations of SLE are highly heterogeneous and commonly involve the skin, musculoskeletal, hematologic, renal, and nervous systems, although the pattern and severity of organ involvement vary considerably between patients [3]. Lupus nephritis is one of the most serious manifestations of SLE and a major cause of morbidity and mortality. Although rare monogenic defects, particularly of early complement components, can cause lupus, most cases of SLE arise through the combined effects of multiple genetic susceptibility loci and environmental triggers [3]. The autoantibody response is directed predominantly against nuclear self-antigens, including DNA, chromatin, histones, ribonucleoproteins (RNP), Sm, and Ro/La antigens. Throughout the present review, SLE refers specifically to the human disease, whereas lupus is used more broadly, particularly in reference to murine models.

Despite major improvements in survival and the introduction of targeted therapies, durable disease control remains difficult, and treatment frequently relies on broad immunosuppression associated with substantial cumulative toxicity [3–5]. Therapeutic development is complicated by the marked clinical and molecular heterogeneity of SLE: patterns of organ involvement and dominant immune pathways vary among patients and may change over the course of disease, while reliable biomarkers for selecting the most appropriate therapy remain limited. This heterogeneity also complicates patient stratification, clinical trial design, and the selection of meaningful endpoints, potentially obscuring therapeutic benefit within biologically responsive subsets and emphasizing the need to define the mechanisms that initiate and sustain autoreactive immune responses.

Autoreactivity is an intrinsic feature of the B cell repertoire [6–8]. In humans and mice, variable (V), diversity (D), and joining (J) gene segment recombination and heavy-light chain pairing generate approximately 10^6^ possible unique B cell receptors (BCRs). Nucleotide insertion and deletion at the segment junctions expand this theoretical repertoire by many additional orders of magnitude, such that it greatly exceeds the approximately 10^8^ B cells in mice and 10^11^ in an adult human [9,10]. While only a small fraction of possible BCRs are represented at any one time, the stochastic nature of receptor recombination permits self-reactive specificities to arise, consistent with their high frequency among newly generated immature B cells [6].

In healthy individuals, these cells are controlled by tolerance checkpoints that eliminate, edit, or functionally restrain autoreactive B cells during early development and after maturation [11–13]. These sequential checkpoints limit the survival, maturation, and activation of self-reactive clones, preventing their incorporation into pathogenic immune responses. Thus, lupus arises when self-reactive clones become activated and enter differentiation pathways that support chronic autoantibody production.

## Self-reactive B cell evolution and epitope spreading in lupus

A defining feature of SLE is that the autoantibody repertoire evolves over time. Early responses are often directed against nuclear antigens and DNA, but the autoimmune response broadens over time to include a wider range of self-antigens. Longitudinal analyses of sera from patients with SLE have shown that new autoantibody specificities appear during disease progression, consistent with ongoing diversification of the autoreactive B cell response [14,15]. At the level of the B cell repertoire, this diversification may arise through recruitment of new autoreactive clones, including clones with limited somatic hypermutation, as well as through continued mutation and selection of clones already participating in the response. The resulting broadening of autoreactivity, termed epitope spreading, contributes to lupus pathogenesis by expanding the range of cellular components and tissues targeted by autoantibodies [2]. Defining the mechanisms that drive this diversification is therefore central to understanding SLE pathogenesis.

## Modelling autoreactive B cell evolution using the 564Igi:WT mixed bone marrow chimera system

Although longitudinal studies in patients demonstrate that autoantibody specificities broaden over time, they cannot easily resolve the mechanisms that drive this diversification. Many murine models of lupus recapitulate B cell activation, autoantibody production, and features of self-reactive B cell evolution, but these responses often arise on complex genetic backgrounds or in settings of broad immune dysregulation. Individual models differ in their underlying genetics, dominant immune pathways, clinical manifestations, and experimental applications, each capturing distinct aspects of human SLE (Table 1) [16–33]. As a result, they do not readily distinguish the dynamics of how an initiating breach in tolerance gives rise to an expanded autoreactive repertoire.

**Table 1:** Representative murine lupus models

Model	Model basis	Principal lupus-like phenotypes	Main experimental applications	Limitations
NZB/W F1 [16–18]	F1 hybrid of NZB and NZW strains; polygenic susceptibility	Female bias; ANA and anti-DNA antibodies; progressive immune complex nephritis	Lupus nephritis; natural disease progression; preclinical therapeutic studies	Relatively late and variable disease onset (commonly around 5-6 months in females); predominantly renal phenotype; requires breeding of parental strains
MRL/lpr [17,19–22]	*Fas^lpr^* loss-of-function mutation on the MRL background	ANA, anti-DNA and anti-Sm/RNP antibodies; nephritis, dermatitis, and arthritis	Extrafollicular autoreactive B cell responses; tissue pathology; Fas-dependent lymphocyte homeostasis; B cell-T cell interactions	Fas deficiency causes marked lymphoproliferation and accumulation of CD4^−^CD8^−^B220^+^ T cells, which are not typical of most human SLE
NZM2410 [23,24]	Recombinant inbred strain derived from NZB and NZW progenitors	ANA and anti-DNA antibodies; spontaneous immune complex nephritis	Polygenic lupus susceptibility; nephritis; genetic mapping; preclinical therapeutic studies	Complex polygenic disease; predominantly renal phenotype
*Sle* congenics [25]	C57BL/6 congenic strains carrying the *Sle1*, *Sle2* and/or *Sle3* susceptibility loci derived from NZM2410	*Sle1*: loss of tolerance; *Sle2*: B cell hyperactivity; *Sle3*: dysregulated T cell activation; combined loci produce systemic autoimmunity and nephritis	Genetic dissection of lupus susceptibility; tolerance checkpoints; analysis of susceptibility loci on a B6 background	Individual loci reproduce only selected components of lupus
*Lyn*^*−/−*^ (26, 27)	Germline *Lyn* deficiency	Dysregulated BCR signaling and B cell tolerance; spontaneous GCs; ANA; immune complex nephritis	BCR signaling; B cell tolerance; GC autoantibody responses	Single-gene signaling defect with B cell-intrinsic and broader immune abnormalities
*Sle1.Yaa* [28]	C57BL/6 congenic males carrying *Sle1* and *Yaa*, a Y-linked duplication of an X-chromosomal interval that includes *Tlr7*	Spontaneous GCs; T follicular helper (Tfh) cell expansion; broad autoantibody responses; nephritis	TLR7 dosage; autoreactive GC biology; Tfh-B cell interactions	Male-specific model; *Yaa* duplicates genes in addition to *Tlr7*
TLR7 gain-of-function [29]	CRISPR knock-in of the orthologous *Tlr7*^Y264H^ gain-of-function substitution identified in a pediatric patient with SLE	ANA and anti-Sm/RNP antibodies; thrombocytopenia; spontaneous GCs; nephritis	TLR7 gain-of-function biology; monogenic lupus	Models a rare, high-penetrance monogenic mechanism and does not capture the genetic heterogeneity of most SLE
*Dnase1l3^−/−^* [30]	Germline *Dnase1l3* deficiency	Anti-DNA and anti-chromatin antibodies; spontaneous autoimmunity	Extracellular chromatin clearance; tolerance to apoptotic microparticle-associated DNA; anti-DNA autoimmunity	Models a rare, high-penetrance monogenic mechanism and does not capture the genetic heterogeneity of most SLE
Pristane-induced lupus [31–33]	Intraperitoneal pristane administration	ANA, anti-DNA, anti-Sm/RNP antibodies; type I IFN signature; nephritis	Environmentally induced lupus; type I IFN and TLR7 biology; inflammatory disease initiation	Artificial induction; strain-dependent susceptibility and variable disease severity

The 564Igi model provides a way to study this process using a defined autoreactive B cell specificity. 564Igi mice carry transgenic immunoglobulin heavy and light chains that together generate an autoreactive BCR recognizing nucleic acid-associated self-antigens, including DNA-, RNA-, and nucleosome-containing complexes [34,35]. This specificity is well suited to engage the dual-signal pathway implicated in lupus, in which BCR-mediated uptake of nucleic acid-containing self-antigens delivers RNA-associated material to endosomal Toll-like receptor 7 (TLR7). Consistent with this idea, loss of tolerance in the 564Igi system is TLR7 dependent, and broader work has shown that B cell-intrinsic TLR7 signalling is required for spontaneous GC formation and the associated loss of B cell tolerance [34,36]. This occurs through RNA-containing self-antigens providing antigen receptor engagement and innate co-stimulation, allowing autoreactive B cells that would otherwise remain restrained to enter pathogenic differentiation pathways [37].

**Figure 1:**
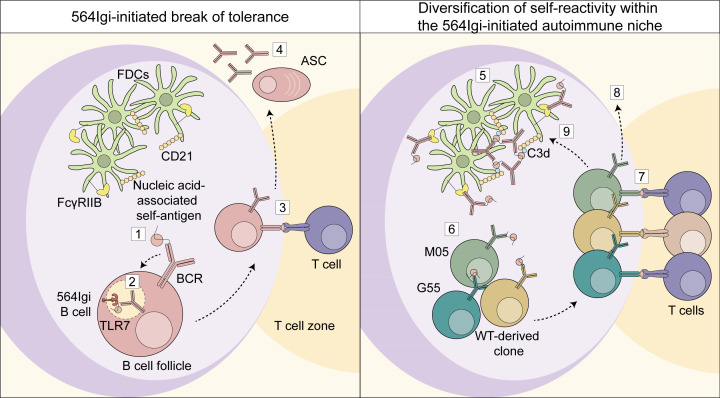
Evolution of self-reactive B cells in the 564Igi autoimmune niche Studies using 564Igi:WT mixed bone marrow chimeras and WT B cell adoptive transfers show how an initiating autoreactive response can recruit otherwise restrained self-reactive B cells into GC and extrafollicular (EF) pathways. 564Igi B cells recognize nucleic acid-associated self-antigens through the BCR (1) and engage TLR7-dependent innate sensing (2). These signals, together with T cell interaction (3), promote activation and antibody-secreting cell (ASC) differentiation (4). The resulting autoantibodies form immune complexes with self-antigen that become complement-tagged and are deposited on follicular dendritic cell (FDC) networks (5). CD21-dependent antigen retention and FcγRIIB-dependent immune complex handling by FDCs help establish stable antigen depots, creating an autoimmune follicular niche that supports recruitment of WT-derived self-reactive clones, including M05 and G55 (6). These recruited clones can receive T cell help (7) and either differentiate through EF pathways toward ASC generation (8) or participate in GC responses, where continued access to FDC-displayed self-antigen (9) supports somatic hypermutation, selection, persistence, and epitope spreading. Together, these pathways link an initiating tolerance breach to chronic diversification of the lupus-like autoantibody response.

When 564Igi bone marrow is mixed with wild-type (WT) bone marrow and transferred into lethally irradiated WT recipients, the resulting mixed bone marrow chimeras develop autoreactive GCs and epitope spreading reminiscent of SLE [38]. Mechanistically, the 564Igi clone breaks tolerance and initiates the autoimmune process, which allows WT B cells to join the response. In 564Igi:WT chimeras, WT B cells dominate the GC response, undergoing clonal expansion and selection to generate a variety of autoantibodies, including to antigens not recognized by 564Igi B cells, such as a proliferation-inducing ligand (APRIL), bactericidal/permeability-increasing protein (BPI), and glomerular basement membrane (GBM) [38]. The present model enables the evolution of a polyclonal WT B cell repertoire to be followed after a defined initiating breach in tolerance. In 564Igi:WT mixed chimeras, 564Igi B cells initiate autoimmunity, but autoreactive GCs become dominated by WT-derived B cells that undergo somatic hypermutation, clonal selection, and epitope spreading. Thus, the system captures how endogenous self-antigen-driven autoimmune responses diversify beyond the initiating autoreactive clone (Figure 1).

The dominance of WT-derived B cells in 564Igi:WT chimeras raised a more mechanistic question—once WT clones have been selected within an autoreactive GC, do they acquire the capacity to initiate autoimmunity themselves, or does their activation remain dependent on the antigenic and inflammatory niche created by the 564Igi clone? To separate BCR specificity from the environment in which that specificity was selected, two autoreactive BCRs derived from WT GC clones in 564Igi:WT chimeras were reconstructed as receptor knock-in mice [39]. B cell clones G55 and M05 represent distinct outcomes of autoreactive repertoire diversification. G55 recognizes small nuclear ribonucleoprotein SmD2, a nuclear antigen targeted in SLE autoantibody responses but not recognized by the initiating 564Igi clone, thereby reflecting epitope-spread autoreactivity. In contrast, M05 recognizes CCL22 and DNA, reflecting convergence toward an autoreactive specificity shared with the initiating 564Igi clone. M05 and G55 knock-in mice did not develop overt GCs or autoantibody production, despite carrying autoreactive BCR sequences. Instead, autoreactivity was restrained primarily through receptor editing, with most mature M05 and G55 B cells expressing λ light chains rather than the inserted κ light chain. However, this tolerance was not absolute. When M05 or G55 bone marrow was combined with 564Igi bone marrow, M05- and G55-derived κ^+^ B cells were selectively enriched within GCs and expressed the inserted heavy chains, indicating that the original autoreactive specificities could be recruited into the autoimmune response. These clones were also functional, producing IgG2c autoantibodies that were detected in serum and deposited in renal glomeruli. These findings show that autoreactive BCR specificity alone is not sufficient to breach tolerance. Instead, pathogenic differentiation depends on the autoimmune context, including self-antigen availability, innate sensing pathways, and T cell help.

A central unresolved question is why some autoreactive clones can create this context, whereas others require it to be established. The comparison between 564Igi and the WT-derived autoreactive clones M05 and G55 provides a useful framework. Although all three BCRs recognize self-antigen, they may occupy different positions in autoimmune evolution. The polyreactive 564Igi BCR may support early EF autoantibody production before self-antigen is stably retained on FDC networks. These autoantibodies could form immune complexes that promote antigen deposition on FDCs and create a GC niche into which additional clones, such as M05 and G55, are recruited. In contrast, M05 and G55 were isolated from WT-derived clones within autoreactive GCs. Their failure to initiate autoimmunity in isolation may therefore reflect GC-biased differentiation. Consistent with the role of BCR signal strength in shaping EF versus GC fate [40], these clones may represent GC-adapted propagating clones that expand the autoreactive repertoire after tolerance has already been breached. This distinction between initiating and propagating clones is consistent with earlier work in the AM14 rheumatoid factor (RF) system, in which autoreactive RF B cells recognize IgG2a [41]. RF B cell activation began focally, correlated with IgG2a-containing immune complexes, and generated ASCs with somatic hypermutation before a clear GC response was established. These findings support an EF-biased route of autoreactive initiation that may create the immune complex-rich niche required for later GC recruitment. This distinction is relevant to lupus models in which GC differentiation is blocked. In 564Igi mice, conditional deletion of B cell lymphoma 6 (Bcl6) in activation-induced cytidine deaminase-expressing B cells blocked GC differentiation but did not prevent anti-DNA autoantibody production or ASC expansion [42]. A related GC-independent phenotype has also been observed in a TLR7 gain-of-function lupus model [29]. These studies show that pathogenic autoantibody production can proceed without GCs but leave open whether this reflects a natural EF trajectory or an alternative fate adopted when GC differentiation is blocked.

## Adoptive transfer approach to study early inclusion of tolerant B cells

A limitation of the mixed bone marrow chimera system is that 564Igi and WT B cells coexist throughout immune reconstitution and subsequent disease progression. This makes it difficult to determine when WT B cells first join the autoimmune response and which signals are required for the recruitment of silent autoreactive clones. In addition, because GC and EF B cell differentiation occur in parallel, the chimera system does not easily distinguish recruitment into autoreactive GCs from GC-independent activation.

To overcome these limitations, we used parabiosis and adoptive transfer approaches to synchronize WT B cell access to pre-existing autoreactive compartments [43]. In parabiosis experiments, WT mice were surgically joined to 564Igi mice, allowing circulating cells to equilibrate. WT B cells entered the 564Igi autoimmune environment and became enriched in the GC compartment of the 564Igi partner. Temporary parabiosis further showed that this recruitment was durable—after surgical separation, WT B cells remained enriched in autoreactive GCs for weeks, indicating that transient exposure to the autoimmune environment was sufficient for WT B cells to enter and persist within the response.

Adoptive transfer provided a more temporally controlled system to define the earliest events of WT B cell recruitment. When isolated mature WT B cells were transferred into 564Igi recipients, donor B cells entered pre-existing autoreactive GCs within days. They were detectable in GCs by day 4 and became enriched in the GC compartment by day 7 relative to their frequency among follicular B cells. Donor-derived autoantibodies were also detectable within the first week after transfer, demonstrating that recruited WT B cells could rapidly participate in the ongoing autoimmune response and contribute to autoantibody production. This entry into autoreactive GCs required BCR specificity, major histocompatibility complex (MHC) class II expression, TLR7, and type I interferon (IFN) receptor signaling, indicating that WT B cells must recognize relevant self-antigen, receive T cell help, and integrate innate nucleic acid-sensing signals to cross the selection barrier into autoreactive GCs. Thus, autoreactive GCs behave as open structures that can recruit new naive B cells, but this recruitment is actively gated by antigen recognition and inflammatory cues.

The same adoptive transfer system also revealed that early WT B cell activation is not limited to the GC pathway. Within days of transfer into 564Igi recipients, donor B cells also generated CD21^lo^CD11c^+^ cells and ASCs, consistent with rapid EF differentiation [44]. This response was strongly TLR7-dependent and was evident at early time points before substantial accumulation of donor B cells within GCs. CD21 down-regulation marked an early activation state with donor CD21^lo^ cells arising from CD21^+^ precursors, and serial transfer experiments showed that CD21^lo^ cells were more biased toward ASC differentiation, whereas cells retaining a follicular phenotype after the initial transfer were more likely to adopt a GC fate. Blocking CD21-ligand engagement impaired donor B cell proliferation and ASC generation, suggesting that complement-tagged self-antigen provides an early CD21-dependent priming signal that cooperates with TLR7 to drive rapid EF-biased autoreactive B cell differentiation.

Together, these studies show that the 564Igi environment supports both GC and EF pathways for WT B cell inclusion. Recruitment into established autoreactive GCs may allow B cells to undergo diversification through somatic hypermutation and selection, whereas EF activation would provide a rapid route to ASC differentiation [45,46]. Consistent with the present model, analysis 6 days after WT B cell adoptive transfer showed increased replacement mutations in the GC compartment compared with the CD21^lo^ B cell compartment, supporting the idea that GCs provide a distinct environment for mutation and selection during autoreactive B cell evolution [44].

Recent work using allelic variants of the immunoglobulin heavy-chain variable (VH) gene IGHV1-69 provides direct evidence that germline-encoded autoreactivity can bias B cell recruitment into autoreactive GCs [47]. The L54 allele of IGHV1-69, but not the closely related F54 allele, encodes polyreactive and autoreactive BCRs. When naive L54/F54 IGHV1-69 B cells with human-like diversity of the complementarity-determining region 3 of the heavy chain were transferred into 564Igi mice, L54 B cells were preferentially retained and enriched in autoimmune GC and ASC compartments. This advantage was lost after transfer into non-autoimmune WT recipients, indicating that the 564Igi environment selectively expands naturally autoreactive B cells. L54 antibodies also showed elevated RNP reactivity, which was reduced by sequential conversion of germline antibodies toward the F54 sequence, supporting a VH-encoded basis for this self-reactivity.

This concept is supported by earlier studies of human B cells carrying the VH4-34 heavy chain variable region, identified by the 9G4 idiotype [48]. In healthy individuals, these inherently autoreactive B cells are present in the naive repertoire but are prevented from entering GCs and are scarce in post-GC IgG memory and ASC compartments [49,50]. In SLE, this checkpoint is defective. 9G4 B cells participate in GCs and are enriched in memory compartments. Thus, lupus-associated GC entry may reflect both clone-intrinsic autoreactivity and disease-specific failure of peripheral censoring. In this context, the 564Igi environment may act analogously to the SLE GC niche, selectively including autoreactive clones that would otherwise remain excluded or functionally restrained.

Together, these findings suggest that naturally self-reactive B cells are poised for recruitment once tolerance is breached, providing a mechanism by which autoimmune GCs can amplify and diversify autoreactive repertoires.

## Regulation of autoreactive B cell inclusion and epitope spreading

The 564Igi:WT chimera model also makes it possible to identify the signals that regulate the evolution of self-reactive B cells in lupus. Autoreactive GC formation, ASC differentiation, and autoantibody deposition are dependent on T cell help, as deficiency of inducible T cell co-stimulator (ICOS) or signaling lymphocytic activation molecule-associated protein (SAP) in 564Igi mice markedly reduces autoreactive GCs, class-switched antibodies, and glomerular autoantibody deposition [51]. Furthermore, tamoxifen-induced deletion of Bcl6 in CD4^+^ T cells on the 564Igi background led to loss of both follicular T cells and established autoreactive GCs, demonstrating that sustained follicular T cell help is required to maintain chronic autoreactive GC responses.

A critical mechanism through which T cell help promotes epitope spreading is linked recognition. B cells with distinct BCR specificities can internalize physically associated components of the same self-antigen and present shared or linked peptides to CD4 T cells. In this way, a limited set of autoreactive T cell specificities provides help to multiple B cell clones, allowing the response to broaden beyond the antigen recognized by the initiating clone. T cell help can therefore act as a bridge between distinct autoreactive B cell specificities, converting an initial breach in tolerance into a diversified GC response.

An important unresolved question is how tolerance is first broken in the autoreactive T cell compartment. One possibility is that autoreactive B cells themselves act as initiating antigen-presenting cells (APCs), capturing self-antigen through the BCR and presenting it efficiently to cognate CD4 T cells. However, T cell priming may also involve myeloid APCs, particularly in an inflammatory environment rich in nucleic acid-containing immune complexes and innate sensing signals. In this model, myeloid APCs would drive the initial activation of autoreactive CD4 T cells, whereas autoreactive B cells subsequently use BCR-mediated antigen capture and presentation to solicit cognate help. Experiments using MHC-mismatched 564Igi:WT chimeras showed that epitope spreading required MHC compatibility between the initiating 564Igi clone and recruited WT B cells [52]. WT B cells that did not share the relevant MHC haplotype of the 564Igi B cells were unable to access autoreactive T cell help, whereas B cells expressing both MHC haplotypes participated in GC responses. Importantly, these dual-MHC ‘bridge’ B cells could compensate for the lack of shared MHC restriction by interacting with T cells from either compartment and relaying autoreactive help to otherwise excluded B cell specificities. Autoantigen array analysis confirmed that the GC response was accompanied by functional epitope spreading at the serological level, with ‘bridge’ chimeras developing broader autoantibody reactivity beyond the initial 564Igi-associated specificity. These findings support a model in which autoreactive B cells can act as APCs to overcome T cell tolerance and relay cognate T cell help, thereby enabling epitope spreading.

Paired single-cell RNA and single-cell T cell receptor (TCR) sequencing has demonstrated that follicular T cells in autoimmune chimeras acquire disease-associated transcriptional programs compared with immunized WT control chimeras [53]. These changes included increased expression of IFN-responsive and activation-associated genes, including Ly6a and Lag3, altered metabolic pathways, and reduced expression of Id3. These transcriptional programs were induced in the absence of global TCR repertoire differences. Nevertheless, a limited subset of clonotypes and predicted specificity groups were preferentially represented in autoimmune compared with immunized WT control chimeras and retained autoimmune-associated transcriptional signatures.

To test how TCR specificity influences autoreactive GC formation and epitope spreading, candidate TCRs were selected from paired scRNA-seq/scTCR-seq analysis of follicular T cells isolated from autoimmune and immunized WT control chimeras [51]. TCR-A and TCR-E were associated with autoimmune 564Igi chimeras, whereas TCR-B was derived from an immunized WT control chimera. Although their precise cognate peptide-MHC ligands were not fully defined, functional reporter assays showed that autoimmune-associated TCRs could respond to antigenic material from 564Igi tissues, supporting the idea that these clonotypes recognize self-antigens generated in the autoimmune setting. When expressed in retrogenic chimeras, a follicular T cell compartment restricted to individual autoimmune-associated TCRs, namely, TCR-A and TCR-E, was sufficient to support autoreactive GC formation and WT B cell entry into GCs, whereas the control TCR-B was not. However, single-TCR chimeras produced narrower and TCR-specific patterns of autoantibody reactivity than chimeras with a diverse WT follicular T cell repertoire. A WT T cell repertoire drove the broadest autoantibody response, including reactivity to the aminoacyl-tRNA synthetases Jo-1 and EJ, and to DNA topoisomerase I, commonly termed Scl-70. Jo-1 and EJ are associated with antisynthetase syndrome, whereas Scl-70 is associated with systemic sclerosis. In the present model, however, their significance is as antigenically distinct targets that demonstrate intermolecular epitope spreading beyond the nucleic acid-associated autoreactivity of the initiating 564Igi clone. Individual autoimmune-associated TCRs promoted more restricted patterns of epitope spreading. TCR-A supported reactivity to Jo-1, Scl-70, and the thyroid autoantigen thyroglobulin, whereas TCR-E supported mainly Jo-1 and Scl-70 reactivity. These findings indicate that T cell help regulates not only whether autoreactive B cells are recruited into GCs, but also which B cell clones are selected and which autoantibody specificities emerge.

Together, these studies suggest a reciprocal model of autoreactive B cell evolution. Autoreactive B cells capture and present self-antigen, thereby activating or sustaining autoreactive follicular T cells; in turn, T cells provide selective help that determines which additional B cell clones enter GCs, persist, and contribute to epitope spreading. In this way, BCR specificity, antigen presentation, and TCR specificity together shape both the breadth and direction of the evolving autoimmune repertoire.

FDC antigen display provides a second layer of regulation. FDCs may regulate autoreactive GC evolution through inhibitory IgG Fc receptor IIB (FcγRIIB)-dependent control of clonal selection. FcγRIIB is up-regulated on FDCs during GC formation, in parallel with the emergence of antigen-specific IgG and immune complexes [54]. In 564Igi:WT mixed bone marrow chimeras, loss of FcγRIIB from the radioresistant stromal compartment does not reduce overall GC B cell frequencies but alters the composition of the response. FcγRIIB deficiency in FDCs results in more clonally diverse autoreactive GCs, including persistence of IgM^+^ clones with lower levels of somatic hypermutation. Thus, FDC FcγRIIB appears to regulate selection stringency once IgG-containing immune complexes accumulate. The mechanism by which this occurs remains unclear. One possibility is that reduced FcγRIIB-dependent immune complex handling by FDCs enhances FcγRIIB-mediated inhibitory signaling in B cells, thereby altering naive B cell activation and GC B cell differentiation. FcγRIIB is an established inhibitory receptor on B cells, and FcγRIIB deficiency in the absence of a lupus-prone background has been shown to promote differentiation of naive B cells toward a GC-like phenotype and to specifically permit the activation and expansion of high-affinity DNA-reactive B cells in anti-DNA BCR transgenic mice [55,56]. Thus, if FDC FcγRIIB normally sequesters or organizes IgG-containing immune complexes on the stromal network, its absence could alter the availability or configuration of these complexes for FcγRIIB co-engagement on B cells. Alternatively, FDC FcγRIIB may shape how IgG-containing immune complexes are displayed to GC B cells, thereby determining whether lower-affinity or less-mutated clones can continue to access antigen and receive sufficient signals to engage and recycle.

Immune complexes and complement-opsonized self-antigens can be retained on FDC networks through CD21/CD35, creating a durable source of antigen within follicles and GCs [57,58]. Because individual interactions between CD21 and the complement component 3d (C3d) are low affinity and rapidly dissociate, stable antigen retention depends on multivalent engagement between multiple CD21 molecules and multiple C3d moieties within immune complexes [58]. In lupus, this mechanism may allow immune complexed self-antigen to persist. This retained antigen could influence autoreactive B cell evolution by making self-antigen available for naive B cell activation and by sustaining antigen availability within established GCs.

Taken together, studies using the 564Igi model have uncovered how autoreactive B cell responses continue to evolve after tolerance is breached. An initially focused response can create an autoimmune environment that recruits otherwise restrained WT B cells into GC and EF pathways, through which clonal expansion, somatic diversification, and differentiation broaden and sustain the autoantibody response. This evolution appears to be shaped by BCR and TLR signaling, T cell-derived cues, and antigen retained within lymphoid niches. The 564Igi mixed bone marrow chimera and adoptive transfer systems are therefore particularly valuable for distinguishing the mechanisms that initiate autoreactivity from those that amplify and maintain an established response. However, the causal roles of GC and EF differentiation, recruitment of additional autoreactive B cell clones, and sustained interactions within lymphoid niches remain to be established. Future work should determine how tolerance is initially breached, why some clones initiate autoreactivity whereas others are recruited only after an autoimmune environment has been established, and which signals determine GC versus EF fate and maintain these responses over time. Defining these checkpoints may reveal opportunities to interrupt the evolution of autoreactive B cell responses before they become persistent and pathogenic.

## Perspectives

- Understanding how self-reactive B cells evolve after tolerance is breached is central to explaining how lupus progresses from limited autoreactivity to a diversified pathogenic autoantibody response.
- Studies using 564Igi mixed bone marrow chimera and adoptive transfer models show that an established autoreactive environment can recruit otherwise restrained WT B cells into GC and EF pathways, providing a framework to define the B cell-intrinsic signals, T cell-derived cues, and follicular niche factors that regulate autoreactive B cell fate.
- Future work should define how tolerance is initially breached in lupus, identify the factors that establish persistent autoreactivity, and determine why some clones initiate this process whereas others require an established autoimmune environment.

## Open Access

Open access for this article is provided via our Subscribe to Open programme, made possible through the support of our subscribing institutions. Learn more here: https://portlandpress.com/pages/open_access_options_and_prices#conditional.

## Abbreviations

ASCs: antibody-secreting cells
APCs: antigen-presenting cells
Bcl6: B cell lymphoma 6
BCRs: B cell receptors
C3d: complement component 3d
D: diversity
EF: extrafollicular
FDC: follicular dendritic cell
GC: germinal center
VH: heavy-chain variable
J: joining
MHC: major histocompatibility complex
RNP: ribonucleoproteins
RF: rheumatoid factor
SLE: systemic lupus erythematosus
TCR: T cell receptor
Tfh: T follicular helper
TLR7: Toll-like receptor 7
IFN: interferon
V: variable
WT: wild-type
